# Cardiovascular Effects of Unilateral Nephrectomy in Living Kidney Donors

**DOI:** 10.1161/HYPERTENSIONAHA.115.06608

**Published:** 2016-01-03

**Authors:** William E. Moody, Charles J. Ferro, Nicola C. Edwards, Colin D. Chue, Erica Lai Sze Lin, Robin J. Taylor, Paul Cockwell, Richard P. Steeds, Jonathan N. Townend, on behalf of the CRIB-Donor Study Investigators

**Affiliations:** From the Birmingham Cardio-Renal Group, Institute of Cardiovascular Science, Departments of Cardiology (W.E.M., N.C.E., C.D.C., E.L.S.L., R.J.T., R.P.S., J.N.T.) and Nephrology (C.J.F., P.C.), Queen Elizabeth Hospital Birmingham and University of Birmingham, Edgbaston, United Kingdom.

**Keywords:** blood pressure, glomerular filtration rate, heart ventricles, left ventricular remodeling, vascular stiffness

## Abstract

**Abstract—:**

There is a robust inverse graded association between glomerular filtration rate (GFR) and cardiovascular risk, but proof of causality is lacking. Emerging data suggest living kidney donation may be associated with increased cardiovascular mortality although the mechanisms are unclear. We hypothesized that the reduction in GFR in living kidney donors is associated with increased left ventricular mass, impaired left ventricular function, and increased aortic stiffness. This was a multicenter, parallel group, blinded end point study of living kidney donors and healthy controls (n=124), conducted from March 2011 to August 2014. The primary outcome was a change in left ventricular mass assessed by magnetic resonance imaging (baseline to 12 months). At 12 months, the decrease in isotopic GFR in donors was −30±12 mL/min/1.73m^2^. In donors compared with controls, there were significant increases in left ventricular mass (+7±10 versus −3±8 g; *P*<0.001) and mass:volume ratio (+0.06±0.12 versus −0.01±0.09 g/mL; *P*<0.01), whereas aortic distensibility (−0.29±1.38 versus +0.28±0.79×10^−3^ mm Hg^−1^; *P*=0.03) and global circumferential strain decreased (−1.1±3.8 versus +0.4±2.4%; *P*=0.04). Donors had greater risks of developing detectable highly sensitive troponin T (odds ratio, 16.2 [95% confidence interval, 2.6–100.1]; *P*<0.01) and microalbuminuria (odds ratio, 3.8 [95% confidence interval, 1.1–12.8]; *P*=0.04). Serum uric acid, parathyroid hormone, fibroblast growth factor-23, and high-sensitivity C-reactive protein all increased significantly. There were no changes in ambulatory blood pressure. Change in GFR was independently associated with change in left ventricular mass (*R*^2^=0.28; *P*=0.01). These findings suggest that reduced GFR should be regarded as an independent causative cardiovascular risk factor.

**Clinical Trial Registration—:**

URL: http://www.clinicaltrials.gov. Unique identifier: NCT01028703.

## Abstract 2

Supplemental Digital Content is available in the text.

Chronic kidney disease (CKD) is highly prevalent and confers an increased risk of cardiovascular disease; at estimated glomerular filtration rate (eGFR) values of <60 mL/min/1.73m^2^, risk increases with a graded relationship to eGFR.^1^ A 30% lower eGFR has been consistently associated with a 20% to 30% higher risk of major vascular events and all-cause mortality.^2^ Whether this relationship is causative remains unclear, and potential mechanisms of disease are still under investigation.^3^ Patients with CKD have a clustering of conventional atherosclerosis risk factors such as hypertension and diabetes, yet these factors perform poorly in risk prediction models.^4,5^ Furthermore, the majority of adverse cardiovascular events in late-stage CKD are not caused by atherosclerotic events such as myocardial infarction, but are attributable to heart failure and sudden cardiac death.^6^ These findings, together with data from imaging and pathophysiological studies, suggest that structural left ventricular (LV) disease and increased arterial stiffness may be the key pathogenic mediators of cardiovascular disease in CKD.^7,8^ However, the numerous coexistent risk factors found in patients with CKD make it difficult to be certain that the reduction in renal function is a causative risk factor.

Kidney donors are ideal subjects to study the effects of a reduction in kidney function on the cardiovascular system. After donation, there is an immediate 50% reduction in GFR followed by a gradual improvement to 60% to 70% of baseline as a result of hypertrophy and hyperfiltration in the remaining kidney.^9^ Nonetheless, at 1-year, eGFR is <60 mL/min/1.73m^2^ in up to one third of subjects.^10,11^ Unlike patients with CKD, however, kidney donors have almost no confounding comorbidity. The Chronic Renal Impairment in Birmingham (CRIB)–Donor study was designed to test the hypothesis that the reduction in renal function in living kidney donors is associated with adverse structural and functional cardiovascular effects.

## Methods

### Study Design

**Figure 1.**
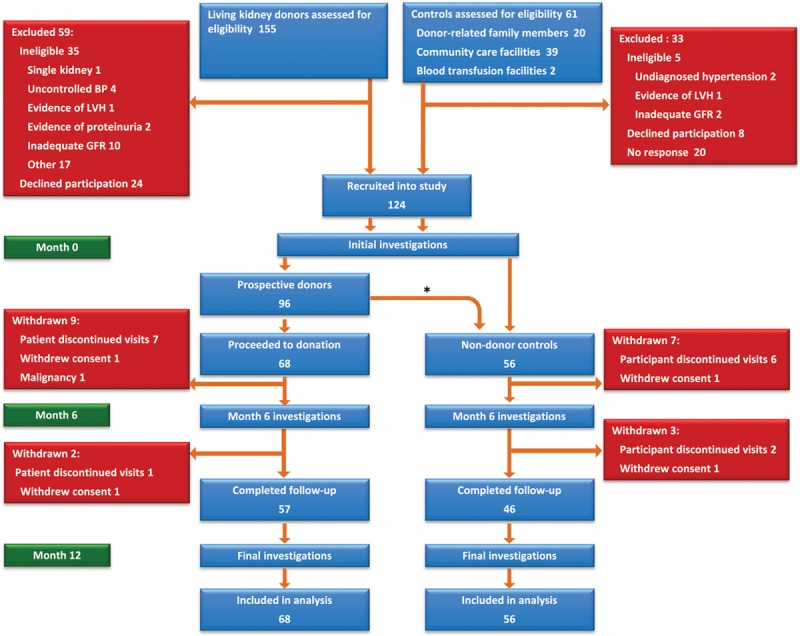
Study timeline. *Twenty-eight participants who did not proceed to living kidney donation were followed as nondonor controls (alternative family member donated [n=12]; recipient health-related issues [n=10]; cadaveric transplant [n=3]; and complicated arterial anatomy [n=3]). BP indicates blood pressure; GFR, glomerular filtration rate; and LVH, left ventricular hypertrophy.

The CRIB-Donor protocol has been reported in detail.^12^ The study had a prospective, longitudinal, parallel group, blinded end point design comparing living kidney donors with similarly healthy controls and was conducted between March 2011 and August 2014 (Figure 1). The study was approved by the West Midlands Research Ethics Committee, adhered to the principles set out by the Declaration of Helsinki, and was registered with the US National Institutes of Health database (NCT01769924). The conduct and reporting of this study were guided by the Strengthening the Reporting of Observational Studies in Epidemiology statement.^13^ All participants gave written informed consent.

### Study Population

Eligible donors were recruited from 2 University hospital–based UK transplant centers. The inclusion and exclusion criteria were identical to those set out in the United Kingdom Guidelines for Living Donor Kidney Transplantation compiled by the Joint Working Party of The British Transplantation Society and The Renal Association (Tables S1 and S2 in the online-only Data Supplement).^14^ To provide a closely matched control population with equivalent baseline health status to the donor cohort, both donors and controls underwent identical screening tests. Control participants were recruited from the same living donor clinics, by identifying individuals who after screening were found to be fit for donation but did not proceed to surgery (eg, because of arterial anatomy, immunological mismatch, or recipient-related health issues). Donor-related family members and subjects attending blood donation and community healthcare facilities who fulfilled medical fitness criteria for living kidney donation were also invited to participate as controls (Figure 1).

### Study Protocol

A flow chart of the study protocol is available in Figure S1. Investigations were performed in each participant at baseline (within 6 weeks before nephrectomy in donors), with subsequent follow-up studies performed at 6 and 12 months. Kidney donors underwent routine follow-up by their local medical and surgical team with no alteration to normal care.

### Outcomes

The primary end point was the change in LV mass at 12 months, as measured by cardiac magnetic resonance imaging (MRI). Secondary end points comprised changes in GFR by isotopic and creatinine-based methods; LV mass:volume ratio by cardiac MRI; office and ambulatory blood pressure; aortic distensibility by MRI; LV strain indices by cardiac MRI feature tracking; urinary albumin:creatinine ratio; high-sensitivity C-reactive protein (hsCRP); serum N-terminal pro-B-type natriuretic peptide (NTpro-BNP), and highly sensitive troponin T. Potential mediators of increased LV mass were also measured; these comprised renin, aldosterone, uric acid, parathyroid hormone (PTH), and fibroblast growth factor-23.

### Cardiac MRI

All studies were performed on a 1.5-T scanner (MAGNETOM Avanto; Siemens, Erlangen, Germany). LV size, function, and mass were assessed using serial contiguous short-axis cine images piloted from the vertical long-axis and horizontal long-axis images of the left and right ventricles (retrospective electrocardiographic gating, SSFP [True-FISP], temporal resolution 40–50 ms, repetition time 3.2 ms, echo time 1.7 ms, flip angle 60°, slice thickness 7 mm with 3-mm gap) in accordance with previously validated methodology.^15^

### Quantification of LV Mass

All LV measurements were made off-line in a Core laboratory by a single observer (W.E.M.) blinded to subject group and temporal sequence (Circle Cardiovascular Imaging Software, cvi42, version 4.2.1, Calgary, Canada). Manual planimetry of the short-axis epicardial and endocardial contours in end-diastole and end-systole was performed using standardized methods.^15^ Mass was calculated as end-diastolic epicardial−endocardial volume×1.05. LV mass was indexed to body surface area using the Mosteller formula. The mass:volume ratio calculated as LV mass/LV end-diastolic volume was used as an index of concentric LV remodeling.^16^

### Assessment of LV Systolic and Diastolic Function

Systolic and diastolic function was assessed using cardiac MRI feature tracking (TomTec 2D Cardiac Performance Analysis, Munich, Germany).^17^ Longitudinal atrioventricular plane displacement was measured in the septum and lateral wall as the distance travelled by the respective annulus from end-diastole to end-systole.^15^ The biplane area–length method was used to measure left atrial volume.^18^

### Aortic Distensibility

Distensibility of the proximal ascending aorta was assessed at the level of the pulmonary artery using the formula: aortic distensibility=Δaortic area/(minimum aortic area×pulse pressure).^19,20^ The measurement of aortic cross-sectional area in systole and diastole was made off-line using automated software (Matlab 6.5; MathWorks Inc., Natick, MA).^21^

### Blood Pressure

Resting blood pressure was measured in triplicate in the nondominant arm using a validated oscillometric sphygmomanometer (Dinamap Procare 200; GE Healthcare, United Kingdom) after 15 minutes of supine rest. The mean of the last 2 brachial blood pressure measurements was used in analysis. Subjects also underwent 24-hour ambulatory blood pressure monitoring (Mobil-O-Graph; IEM GmbH, Stolberg, Germany).

### Measurement of Carotid Intima-Media Thickness

Right and left common carotid arteries were imaged using high-resolution ultrasound (Philips iE33, L9-3 MHz linear array transducer) 1 cm proximal to the carotid bifurcation in accordance with published guidelines.^22^ Common carotid intima-media thickness was measured as the distance between lumen-intima and media-adventitia interfaces using automated wall tracking software (IMT plug-in, QLAB). The mean of the right and left carotid measurements was used for analysis.

### Assessment of Kidney Function

Isotopic GFR (iGFR) was performed at baseline and 12 months in donors and controls using the renal clearance of 51Cr-EDTA.^23^ The CKD-EPI (Epidemiology Collaboration) equation was used to determine eGFR_cr_ with serum creatinine recalibrated to be traceable to an isotope-derived mass spectroscopy method.^24^ Urinary albumin:creatinine ratio was determined from a spot morning urine sample.^25^ Moderately increased albuminuria (category A2, formerly microalbuminuria) was defined by an albumin:creatinine ratio of 30 to 300 mg/g.^26^ The methods used for biomarker assessment are available in the online-only Data Supplement.

### Reproducibility

To assess intraobserver report variability of LV mass measurement, 10 baseline studies were reanalyzed by the same observer (W.E.M) 4 weeks later, blinded to the original data. A random subset of participants (n=10) also underwent repeat imaging within 7 days to determine interstudy reproducibility; both scans were analyzed by the same observer (W.E.M) blinded to temporal sequence and patient identity. Reliability was assessed using the intraclass correlation coefficient with a model of absolute agreement.

### Sample Size Determination

Sample size calculation was based on the primary outcome of change in LV mass. Assuming an SD of change in LV mass of 12 g based on our own data from a previous intervention study in CKD,^27^ a sample size of 55 subjects per group provided 90% power to detect a change in LV mass of 7.5 g with a α=0.05. Therefore, we aimed to recruit 70 patients per group to allow for potential loss to follow-up.

### Statistical Analysis

Data are expressed as mean±SD, median (interquartile range), or frequency (%), unless otherwise stated. The normality of distribution for continuous variables was determined using the Kolmogorov–Smirnov test. Variables not normally distributed were log-transformed. Baseline data were analyzed using independent samples Student *t* or Fisher exact tests. The primary analysis tested differences between groups >12 months using repeated-measures ANOVA. A further quantitative analysis was performed using a last observation carried forward principle. Generalized estimating equations were used to compare donors with controls for the likelihood of developing detectable serum and urinary biomarkers >12 months. Independent predictors of changes in LV mass were determined using multivariable logistic regression. To allow for multiple comparisons, *P* values were adjusted using the Holm–Bonferroni method. A 2-tailed *P*<0.05 was considered statistically significant.

## Results

### Study Participants

A total of 124 subjects were enrolled (68 donors and 56 controls). The first participant entered the study in March 2011 and recruitment continued until August 2013 with the last follow-up visit in August 2014. Because of resource constraints, recruitment was terminated before reaching our prespecified target (140) but after attaining adequate numbers with 12-month follow-up data to provide >85% power to detect the specified change in LV mass. Baseline characteristics of the 2 study groups are shown in Table 1. There were no significant differences between groups. Three donors (5%) were on antihypertensive therapy at baseline. For all participants, blood pressure was <140/90 mm Hg.

**Table 1.**
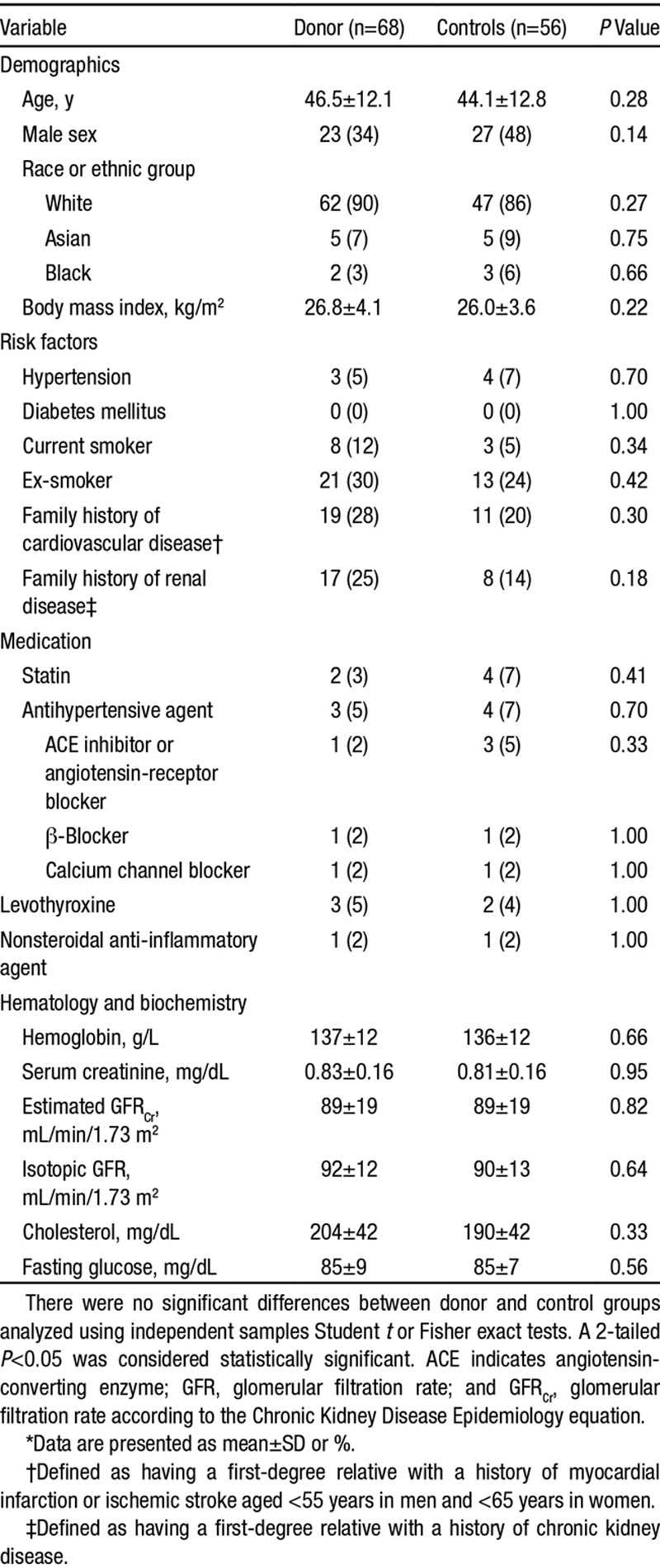
Demographic and Clinical Characteristics at Vaseline*

### Follow-Up and Events

Eleven donors did not complete follow-up to 12 months (Figure 1); 2 declined continued participation because of the travelling involved, 8 could not be contacted, and 1 was withdrawn after diagnosis of a malignancy. No subjects died or had a cardiovascular event during the study.

### Change in Kidney Function and Biochemical Effects

The changes in biochemical indices and cardiovascular disease biomarkers are presented in Table 2. There was a mean decrease in iGFR in donors of −30±12 mL/min/1.73m^2^ and no clinically significant change in controls (−1±10 mL/min/1.73m^2^; *P*<0.001). At 12 months, over one third of donors (35%) had an iGFR <60 mL/min/1.73m^2^, whereas more than one half (53%) had an eGFR <60 mL/min/1.73m^2^. When compared with controls, donors had significant increases in uric acid (+0.94±0.59 versus +0.03±0.55 mg/dL; *P*<0.001), iPTH (+1.1±1.6 versus +0.4±1.3 pg/mL; *P*=0.03), fibroblast growth factor-23 (+18 [+7 to +26] versus +3 [−7 to +17] RU/mL; *P*=0.046), and hsCRP (+1.7±5.3 versus −0.7±5.2 mg/dL; *P*<0.01). Detectable highly sensitive troponin T (odds ratio, 16.2 [95% confidence interval, 2.6–100.1]; *P*<0.01) and microalbuminuria (odds ratio, 3.74 [95% confidence interval, 1.09–12.75]; *P*=0.04) became significantly more common in donors than in controls. There were no significant changes in levels of renin or aldosterone.

**Table 2.**
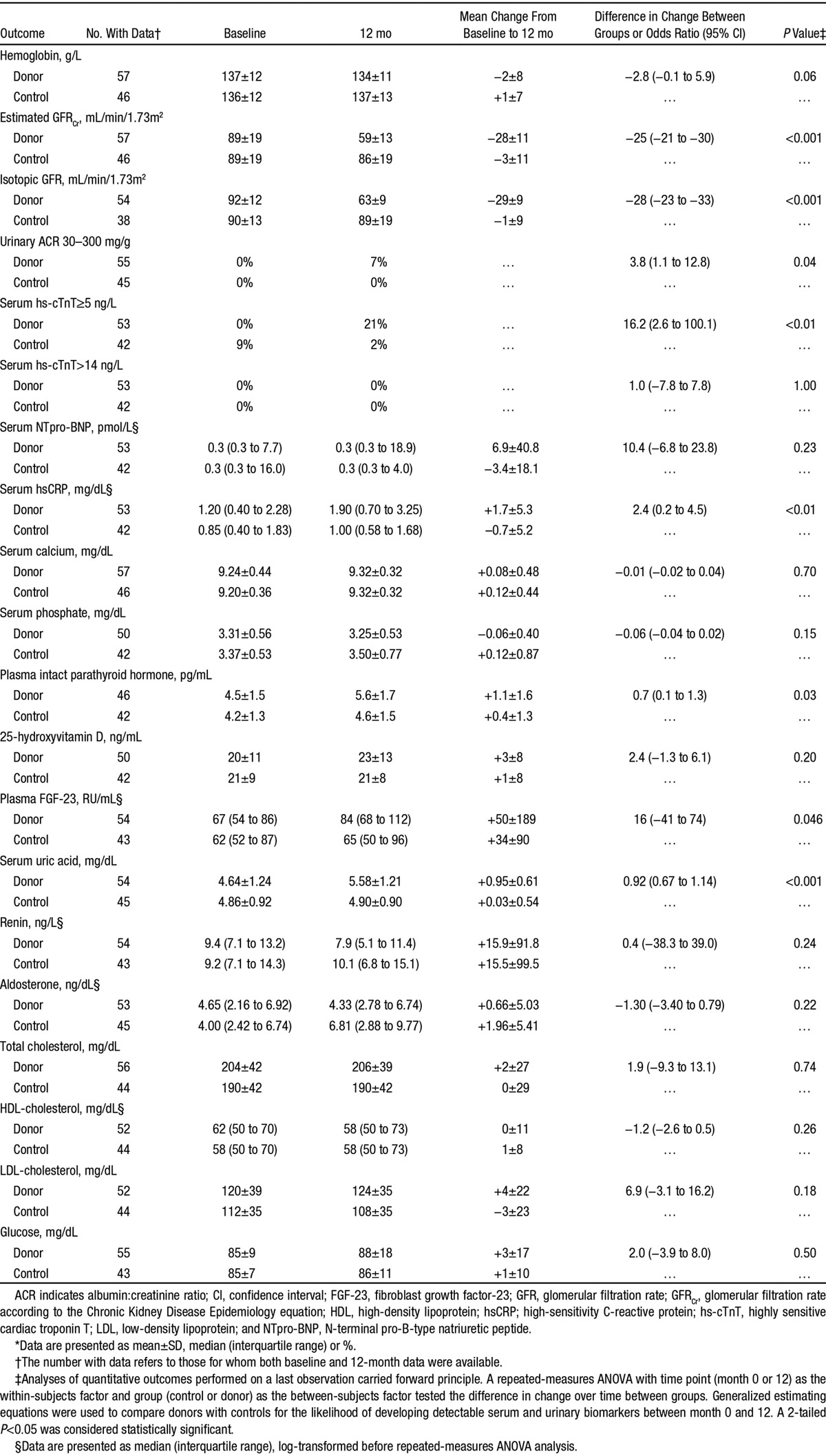
Hematological and Biochemical Effects of a Reduction in Kidney Function*

### Effects on LV Mass, Volumes, and Function

**Figure 2.**
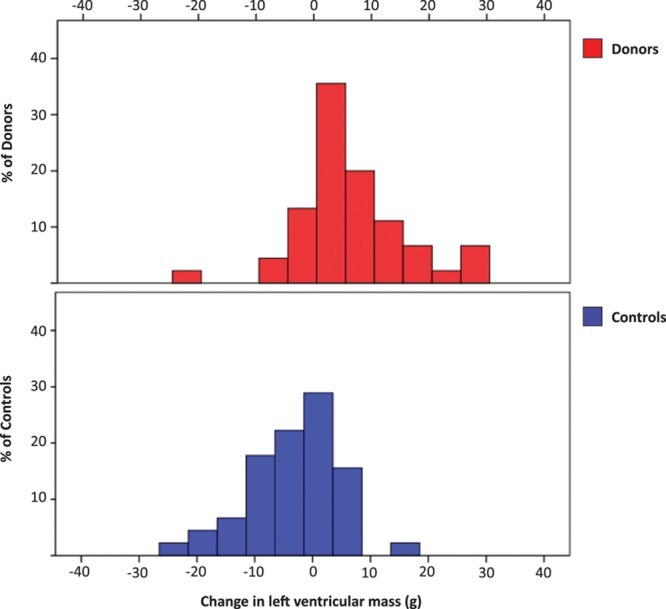
Spread of 12-mo change in left ventricular mass among donors vs controls. There was a significant increase in left ventricular mass in donors vs controls at 12 mo with a mean difference in the change >12 mo of 9.8 g (95% confidence interval, 6.2–13.3; *P*<0.001).

The effects of nephrectomy on cardiac structure and function are presented in Table 3. LV mass increased significantly in donors when compared with controls (+7±10 versus −3±8 g; *P*<0.001; Figure 2). There were corresponding increases in LV mass index (+3.5±5.0 versus −1.6±4.3 g/m^2^; *P*<0.001) and LV mass:volume ratio (+0.06±0.12 versus −0.01±0.09 g/mL; *P*<0.01). Nephrectomy did not affect LV volumes or ejection fraction but was associated with a reduction in long-axis function determined by septal AV plane displacement compared with controls (−1±2 versus 0±2 mm; *P*=0.03). LV systolic function as measured by global circumferential strain was reduced with directionally similar but nonsignificant changes in global longitudinal strain. There was no difference in mean left atrial volume.

**Table 3.**
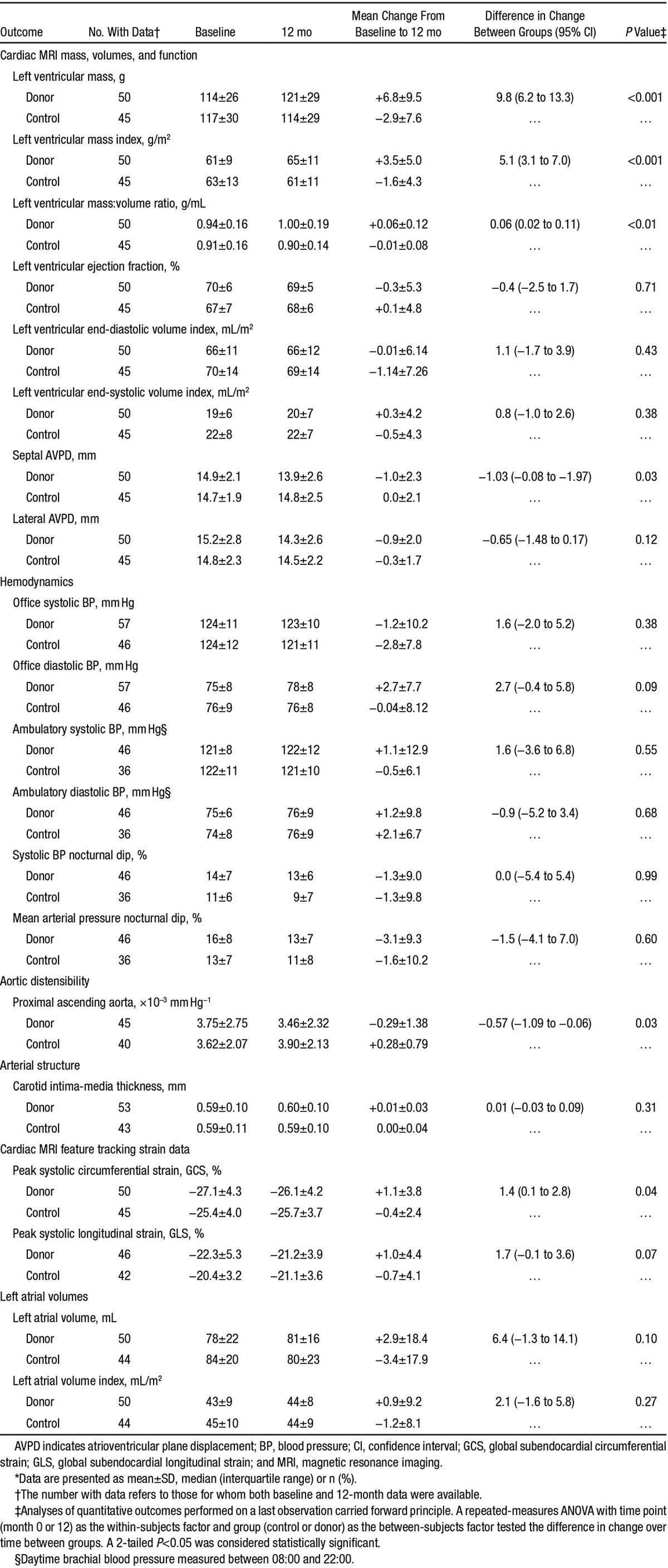
Cardiovascular Effects of a Reduction in Kidney Function*

### Effects on Hemodynamics and Arterial Stiffness and Structure

In donors, there was a significant reduction in aortic distensibility at 12 months post nephrectomy (−0.29±1.38×10^–3^ versus +0.28±0.79×10^–3^ mm Hg^−1^; *P*=0.03). There were no significant differences between donors and controls in any office or ambulatory measures of blood pressure (Table 3). There was no change in carotid intima-media thickness in donors or controls.

### Determinants of LV Mass

Univariate analysis showed that there was an association between the baseline LV mass and the iGFR (β=0.3; *R*^2^=0.09; *P*=0.007). There was also an association between the increase in LV mass and the decrease in iGFR (β=−0.3; *R*^2^=0.19; *P*<0.001). This relationship remained significant after adjustment for age, sex, baseline LV mass, and 12-month changes in blood pressure, uric acid, iPTH, and fibroblast growth factor-23 (β=−0.3; *R*^2^=0.28; *P*=0.01).

### Reproducibility

Intraobserver and interstudy variability for LV mass was low (intraclass correlation coefficient [95% confidence interval], 0.99 [0.98–1.00] and 0.98 [0.91–1.00], respectively).

## Discussion

This study has demonstrated that unilateral nephrectomy in healthy subjects is associated with structural and functional cardiovascular abnormalities within 1 year. There was an increase in LV mass, which had a graded independent association with the reduction in GFR after adjustment for demographic and hemodynamic factors. In addition, there was reduced LV myocardial deformation and increased aortic stiffness. There was no effect on atherosclerosis progression as measured by carotid intima-media thickness. The prevalence of microalbuminuria and detectable cardiac troponin increased after donation, and there was a significant rise in hsCRP. These findings provide insight into the pathophysiological effects of CKD on the cardiovascular system, suggesting that they are not primarily atherosclerotic but instead mediated via myocardial hypertrophy and increased large artery stiffness.

Despite robust epidemiological evidence of an association between reduced eGFR and increased cardiovascular risk,^1^ clear proof of causation has been lacking. The debate has been heightened by an increasing body of opinion, suggesting that early stage CKD is benign and that age-related nephron loss should not be medicalized.^28^ Distinguishing causality from association in CKD is made difficult by the numerous concomitant diseases that accompany reduced GFR including hypertension, diabetes mellitus, anemia, and dyslipidemia. By studying kidney donors, we were able to examine prospectively the effect of a reduction in kidney function on the cardiovascular system, free from such confounding disease processes. The results suggest that reduced GFR is an independent graded risk factor for adverse LV remodeling independent of blood pressure. Because the prevalence of early stage CKD is high (1 in 7 people in the United States),^29^ it may be a major cause of increased LV mass in the community, a potent risk factor for cardiovascular death and adverse events.^30^ Further investigation is required to identify the mediators of renal cardiovascular disease. We have found no evidence to support effects mediated by blood pressure or activation of the renin–angiotensin system. Uric acid, PTH, and fibroblast growth factor-23 all require further investigation as mediators of both increased ventricular mass and arterial stiffening.^31,32^

Our findings may have clinical implications as all of the variables examined are of prognostic importance. Both LV mass and aortic stiffness have been consistently associated with increased mortality in both the general population^16,33^ and in patients with CKD.^8,34^ Although no subject in the current study reached criteria for LV hypertrophy, both LV mass and mass:volume ratio have continuous rather than dichotomous relationships with risk.^35^ Indeed, LV mass is second only to age in its ability to predict cardiovascular events, cardiovascular death, and total mortality.^36^ Detectable troponin also independently predicts both total and cardiovascular mortality in healthy adults,^37,38^ and adverse cardiovascular events in subjects with CKD.^39^ Furthermore, there is a strong independent association between hsCRP and mortality in healthy subjects, as well as in the nondialysis CKD population.^40,41^ The lack of an increase in circulating NTpro-BNP (normally secreted in response to increased wall tension) is perhaps unexpected given the degree of adverse LV remodeling, but may be the result of a compensatory increase in wall thickness normalizing wall stress.^42^ When considering the clinical implications of our findings, it should be noted that few donors reached an eGFR <45 mL/min/1.73m^2^, a level at which excess cardiovascular risk rises sharply.^43^

The practice of living kidney donation is expanding rapidly; to date >135 000 such procedures have been performed in the United States alone.^44^ Although observational studies of kidney donors have been reassuring with adverse event rates lower than those of the general population,^45,46^ studies using highly selected healthy control groups suggest that there may be a small increase in the risk of cardiovascular events and in the risk of developing end-stage CKD, particularly in non-white ethnic groups.^47,48^ Our results provide insight into the potential mechanisms by which adverse cardiovascular effects of kidney donation may be mediated and extend those which have been presented in the only other prospective, controlled pathophysiological study of kidney donors to date.^49^ Kasiske et al^49^ also found no effect of kidney donation on peripheral blood pressure, but recorded similar biochemical effects including an increase in PTH and uric acid. Both these factors have been associated with adverse cardiovascular outcomes and may themselves have direct adverse effects on LV geometry.^50,51^ The lack of change in hsCRP in donors reported by Kasiske et al^49^ is in contrast to our findings.^47^ Our own donor cohort had a higher mean age (47±12 versus 43±12 years) and included twice the proportion of non-white subjects (10% versus 5%), which could explain this discrepancy.

The strengths of our study include its prospective blinded end point design, the selection of similar donors and controls, and the high recruitment rate, but there are some limitations. First, the study was not randomized, which may have introduced bias although randomized trials of donation would never be possible for ethical reasons. Second, although over one half of all subjects approached (57%) took part in the study, we cannot exclude the potential for selection bias. Third, the use of nonsurgical controls means that we cannot exclude the possibility that other factors related to surgery may have exerted adverse cardiovascular effects. Fourth, most donors included were white (90%) and whether similar adverse changes in LV structure and function occur in non-white donors requires further assessment. Finally, our study was not large enough, or of sufficient duration to provide data on the risk of adverse outcomes.

## Perspectives

Compared with healthy controls, kidney donors exhibited adverse changes in LV mass and systolic function, aortic stiffness, and cardiovascular disease biomarker profile at 12 months after donation. The increase in LV mass was independently related to changes in kidney function but independent of blood pressure. These findings suggest that reduced renal function should be regarded as an independent causative cardiovascular risk factor.

## Acknowledgments

This article has been prepared on behalf of the Chronic Renal Impairment in Birmingham (CRIB)-Donor study investigators. We are grateful to the following nurses for help with participant visits: Theresa Brady, Donna Begg, Kathryn Adams, and Elizabeth Dwenger. We are grateful to Dr Daniel Zehnder for facilitating access to subjects undergoing transplant at University Hospital Coventry and Warwickshire. We are also indebted to the Oxford CMR group (Paul Leeson, Matthew Robson, and Adam Lewandowski) for help in setting up the automated analysis of aortic distensibility. Finally, we wish to thank James Hodson and Peter Nightingale for help with the statistical analysis.

## Sources of Funding

W.E. Moody was supported by a British Heart Foundation Clinical Research Fellowship (FS/11/17/28700). The study was performed in the National Institute for Health Research/Wellcome Clinical Research Facility and was also supported by a Queen Elizabeth Hospital Birmingham Charity Grant.

## Disclosures

None.

## Supplementary Material

## Novelty and Significance

### What Is New?

- Even early stage chronic kidney disease is associated with an increased risk of cardiovascular morbidity and mortality, but it has been difficult to know whether this is caused directly by reduced renal function or results from the clustering of risk factors that invariably accompany chronic kidney disease, such as hypertension and diabetes mellitus. We have shown that the modest reduction in renal function caused by kidney donation is associated with increased left ventricular mass and increased arterial stiffness without a change in blood pressure.

### What Is Relevant?

- Reduced renal function is associated with adverse left ventricular remodeling. As mild chronic kidney disease is common, it may be a frequent cause of left ventricular hypertrophy and elevated cardiovascular risk in the community. Kidney donation may confer elevated cardiovascular risk although this is still likely to be less than that of the general population.

### Summary

This study provides strong evidence that a reduction in glomerular filtration rate is independently associated with adverse effects on the myocardium and large arteries.
