# Supplementary material for: Cardiovascular Effects of Unilateral Nephrectomy in Living Kidney Donors
Source: Hypertension. 2016 Jan 3;67(2):368–77. doi: 10.1161/HYPERTENSIONAHA.115.06608 (PMC4716285; doi:10.1161/HYPERTENSIONAHA.115.06608)
Supplement: Supplementary file 1 [file hyp-67-368-s001.docx]

**ONLINE SUPPLEMENT**

**Cardiovascular Effects of Unilateral Nephrectomy in Living Kidney Donors**

William E. Moody, BMedSc, MRCP, Charles J. Ferro, MD, FRCP, Nicola C. Edwards, PhD, MRCP; Colin D. Chue, PhD, MRCP; Erica Lai Sze Lin, BMedSc, MBChB, Robin J. Taylor, MRCP, Paul Cockwell, PhD, FRCP, Richard P. Steeds, MD, MA, FRCP, Jonathan N. Townend, BSc, MD, FRCP.^†^

*On behalf of the CRIB-Donor study investigators*

^†^Address for correspondence: Professor Jonathan N. Townend, Birmingham Cardio-Renal Group, Centre for Clinical Cardiovascular Science, Nuffield House, Queen Elizabeth Hospital Birmingham, Edgbaston, B15 2TH, United Kingdom.

Email: john.townend@uhb.nhs.uk

Tel: +44 (0)121 371 4623

Fax: +44 (0)121 371 4629

Web: [*www.birmingham.ac.uk/bcrg*](http://www.birmingham.ac.uk/bcrg)

Institution address for all authors: Birmingham Cardio-Renal Group, Centre for Clinical Cardiovascular Science, Nuffield House, Queen Elizabeth Hospital Birmingham, Edgbaston, B15 2TH, United Kingdom.

**SUPPLEMENTAL TEXT**

**Biomarker assessment**

Highly sensitive Troponin T was measured by a sandwich principle immunoassay (cobas 8000 modular analyzer, Roche Diagnostics, Burgess Hill), with a lower limit of detection of 5 ng/L and a 99th percentile value in apparently healthy individuals of 14 ng/L.[^1^](#_ENREF_1) Serum NT-proBNP was measured by sandwich immunoassay with magnetic particle separation and chemiluminescent detection (Roche Diagnostics, Burgess Hill) with a lower limit of detection of 0.6 pmol/L.[^2^](#_ENREF_2) Serum C-reactive protein was measured using a highly sensitive assay by latex-enhanced nephelometry (Full Range CRP, SPA_PLUS_ analyser, The Binding Site Group Ltd, Birmingham, UK). Plasma intact PTH was measured by a sandwich immunoassay method (Roche Diagnostics, reference range, 3.5-6.5 pmol/L). Plasma FGF-23 was measured using second-generation C-terminal human FGF-23 ELISA kits (Immutopics, San Clemente, CA).[^3^](#_ENREF_3) Plasma 25-hydroxyvitamin D was measured by liquid chromatomography tandem mass spectrometry. Renin was measured using the Cis-Bio immunoradiometric (IRMA) kit (Codolet, France) and aldosterone was determined by tandem mass spectrometry on an AB Sciex 6500 triple quad mass spectrometer coupled to a Shimadzu Nexera XR UPLC (Warrington, UK). Serum calcium, phosphate and uric acid were measured by standard automated methods.

**SUPPLEMENTAL REFERENCES**

1. Giannitsis E, Kurz K, Hallermayer K, Jarausch J, Jaffe AS, Katus HA. Analytical validation of a high-sensitivity cardiac troponin t assay. *Clin Chem*. 2010;56:254-261.

2. Downie PF, Talwar S, Squire IB, Davies JE, Barnett DB, Ng LL. Assessment of the stability of n-terminal pro-brain natriuretic peptide in vitro: Implications for assessment of left ventricular dysfunction. *Clin Sci (Lond)*. 1999;97:255-258.

3. Smith ER, Cai MM, McMahon LP, Holt SG. Biological variability of plasma intact and c-terminal FGF23 measurements. *J Clin Endocrinol Metab*. 2012;97:3357-3365.

**SUPPLEMENTAL TABLES**

**Table S1. Study inclusion and exclusion criteria.**

**Inclusion criteria**

Age 18-80 yrs

Acceptable GFR by donor age prior to donation^*^

**Exclusion criteria**

Hypertensive end-organ damage, uncontrolled hypertension or the requirement for more than 2 anti-hypertensive medications

Significant proteinuria^†^

Left ventricular dysfunction

Diabetes mellitus

Atrial fibrillation

Any history of cardiovascular or pulmonary disease that would preclude kidney donation

^*^Based on the anticipation of having a GFR of >37.5 mL/min/1.73m^2^ aged 80 years (see Table below).

^†^Urinary albumin–creatinine ratio >300 mg/g, protein–creatinine ratio >500 mg/g or 24-hour total protein >300 mg/day.

GFR, glomerular filtration rate.

**Table S2. Acceptable baseline GFR by age.**

| **Donor age**  **(years)** | **Acceptable corrected GFR**  **(ml/min/1.73m^2^)** |
| --- | --- |
| 18 to 46 | 80 |
| 50 | 77 |
| 60 | 68 |
| 70 | 59 |
| 80 | 50 |

GFR, glomerular filtration rate.

**SUPPLEMENTAL FIGURE**

**Figure S1. Study protocol.**

**
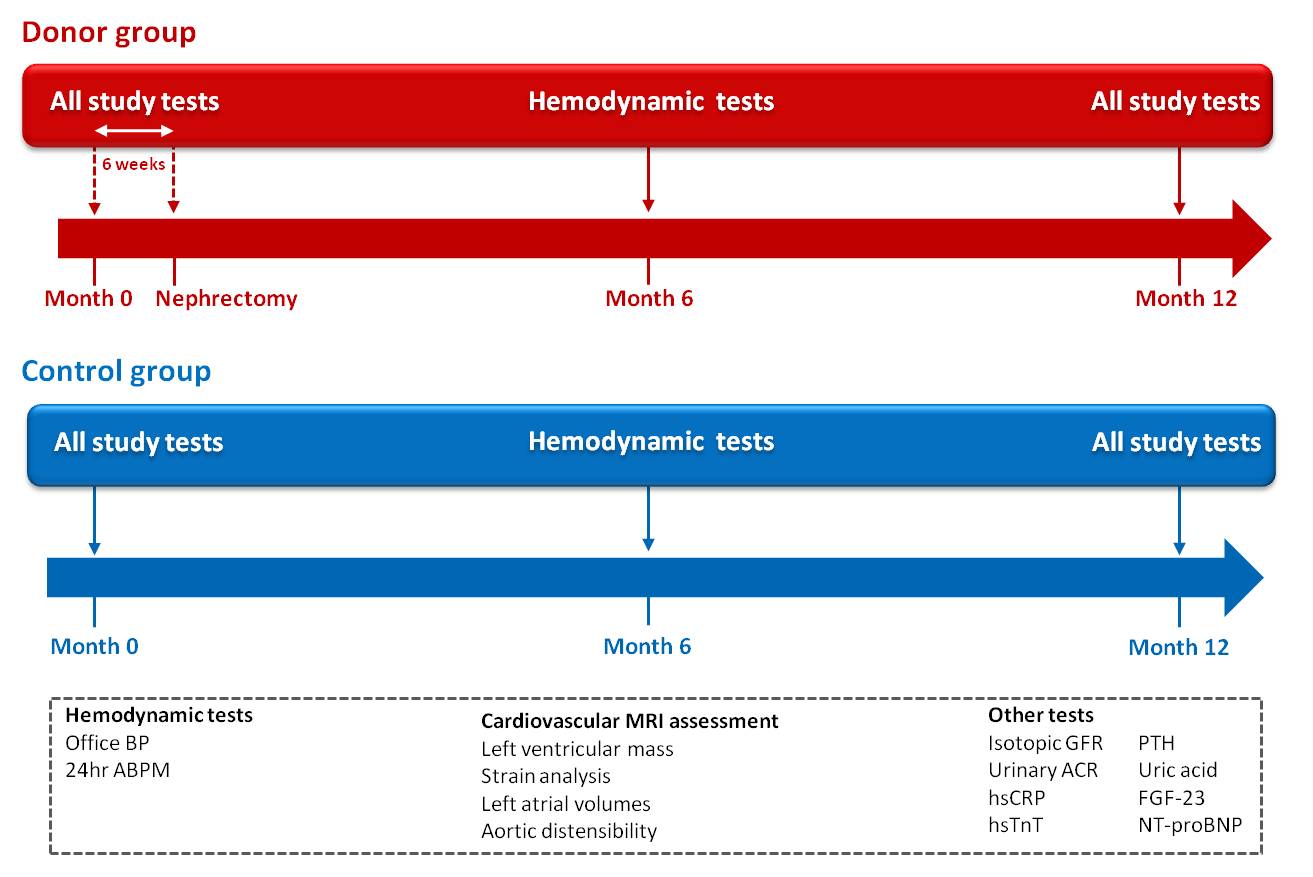
**

ACR, albumin–creatinine ratio; ABPM, ambulatory blood pressure monitor; BP, blood pressure; FGF-23, fibroblast growth factor-23; GFR, glomerular filtration rate; hsCRP, high sensitivity C-reactive protein; hsTnT, highly sensitive Troponin T; LV left ventricular; NT-proBNP, N-terminal pro B Natriuretic peptide; PTH; parathyroid hormone.
